# Improvement of Bending Stiffness of Timber Beams with Ultra-High-Modulus-Carbon-Fibre-Reinforced Polymer Sheets

**DOI:** 10.3390/ma18010071

**Published:** 2024-12-27

**Authors:** Michał Marcin Bakalarz, Paweł Grzegorz Kossakowski

**Affiliations:** Department of Theory of Structures and Building Information Modeling (BIM), Faculty of Civil Engineering and Architecture, Kielce University of Technology, Al. Tysiaclecia Panstwa Polskiego 7, 25-314 Kielce, Poland; mbakalarz@tu.kielce.pl

**Keywords:** bending, composite beam, ductility, numerical modelling, reinforcement, stiffness, wood structures

## Abstract

The bending stiffness of beams represents a pivotal parameter influencing both the dimensions of the elements during their design and their subsequent utilisation. It is evident that excessive deflections can cause discomfort to users and contribute to further structural degradation. The objective of this study was to enhance the bending stiffness of timber beams by bonding a composite sheet to their external surfaces. A carbon sheet exhibiting an ultra-high modulus of elasticity and low elongation at rupture was employed. Two variables of analysis can be distinguished including whether the reinforcement was applied or not and the number of reinforcement layers. The beams, with nominal dimensions of 80 × 80 × 1600 mm, were subjected to a four-point bending test in order to ascertain their mechanical properties. In total, 15 beams were tested (5 unreinforced and 10 reinforced). The reinforcement had no appreciable impact on the increase in flexural load capacity, with the maximum average increase recorded at 9%. Nevertheless, an increase in stiffness of 34% was observed. Additionally, significant increases were observed in ductility up to 248%. However, the ductile behaviour of the beam occurred after the rupture of the reinforcement. In all instances, the failure was attributed to the fracturing of the wooden components or the UHM CFRP (ultra-high-modulus-carbon-fibre-reinforced polymer) sheet. The numerical analysis proved to be a valuable tool for predicting the stiffness of the wood–composite system, with a relatively low error margin of a few percentage points. The modified approach, based on the equivalent cross-section method, permits the determination of a bilinear load deflection relationship for reinforced beams. The aforementioned curve is indicative of the actual behaviour. Given the propensity for the sudden rupture of reinforcement, the described method of reinforcement is recommended for beams subjected to lower levels of stress.

## 1. Introduction

In selecting a composite material for the purpose of reinforcing a timber structure, it is of paramount importance to consider three key parameters in terms of the mechanical properties of the material in question. The aforementioned properties encompass the modulus of elasticity, tensile strength, and elongation of the composite at failure.

It is the view of the authors that tensile strength is a relevant consideration when using small thickness reinforcement in the form of sheets, fabrics, or meshes. Such reinforcement can be described as surface reinforcement, which serves to bind the defects in the wood that are exposed at the surface. In the case of solid reinforcement, defined as materials with larger cross-sectional dimensions and bar-like characteristics, such as bars, sections, plates, and so forth, this parameter assumes a secondary importance. It is, of course, linked to the possibility of fully exploiting its properties and, thus, optimising material consumption.

It can be reasonably deduced that surface reinforcement, with a minimal degree of reinforcement, will ultimately fail in conjunction with the material in question. The observed destruction may be attributed to the attainment of the material’s tensile strength or the ramifications of fracturing within the timber structure. This phenomenon can be observed in a multitude of scientific works. For timber beams reinforced with CFRP (carbon-fibre-reinforced polymer) sheets (one and two layers), Yeou-Fong Li et al. [1] reported a form of failure resulting from composite rupture and wood cracking. Kossakowski achieved this result in a paper on the strengthening of beams with aramid sheets [2]. The same behaviour was described by Sokolowski and Kossakowski [3] for the strengthening of timber beams with FRCM-PBO (fibre-reinforced cementitious matrix–p-phenylene benzobis oxazole) meshes. Analogous results were presented for full-sized wooden and laminated veneer lumber beams reinforced with one layer of a CFRP sheet [4]. It is, therefore, recommended that materials exhibiting the highest possible tensile strength be employed in such instances. Alternatively, the failure of reinforcement can be prevented by increasing the number of layers applied globally or locally.

The utilisation of reinforcement with greater cross-section dimensions (rods, plates, etc.) will probably result in the exploitation of their strength to a maximum of a few tens of percent. It is, thus, possible to utilise reinforcement materials with inferior parameters. In such instances, the timber or bond is destroyed, while the composite reinforcement remains intact. Nowak reported these findings, as did Jasienka et al. [5,6], when reinforcing solid beams with CFRP laminates and steel sections.

The modulus of elasticity of the composite material exerts a markedly greater influence on the performance of reinforced beams. This is due to the fact that it affects the functioning of the wood–composite system from the outset. A significant number of works place considerable emphasis on the importance of the ratios of the modulus of elasticity of the reinforcing material and the reinforcing element [7,8]. An elevated ratio indicates a more efficacious reinforcement. In this context, regardless of the configuration of the composite reinforcement, the most robust materials should be employed. Andor and Bellovics [9] analysed changes in the stiffness of Norway spruce beams unreinforced and reinforced with CFRP fabric in the function of load. The authors concluded that the modulus of elasticity of the beams changes during tests. The effectiveness of beam stiffness increases with the increase in the grammage of the fabrics [10]. Similarly, it increases with the number of applied layers of sheets [11].

In contrast, the phenomenon of elongation at failure exerts an influence on the overall ductility of the system. As the elongation at failure increases, the maximum deformation of the wood also increases. The process of plasticisation can occur in both the tension and compression zones of the wood.

In conclusion, when selecting an optimal material for the reinforcement of timber structures, the following factors should be taken into account (in the preliminary design stage): (1) select a material with the smallest possible cross-section to ensure an optimal match between wear and tensile strength; (2) always select a material with the highest possible elastic modulus; and (3) utilise materials whose limit of elongation at failure will exceed the anticipated maximum deformation of the component to be strengthened.

This paper presents an analysis of the static performance of unreinforced and sheet-reinforced CFRP UHM (ultra-high modulus) timber beams. The material meets two of the three aforementioned prerequisites. The material displays a disadvantage in that it exhibits a low elongation value at failure. The objective of this study is to ascertain the efficacy of reinforcing timber beams in terms of bending stiffness. The second section sets forth the principal guidelines for the methodology of the test and the materials employed. In the third section, the principal findings are presented with reference to the work of other researchers in the field. The final section presents the principal findings of the research project.

## 2. Materials and Methods

### 2.1. Materials

The materials selected for use in the experimental studies were chosen on the basis of their availability and popularity on the Polish market. It is reasonable to posit that the aforementioned assumption will facilitate the utilisation of the extant solutions within the context of engineering practice.

#### 2.1.1. Wood

This study employed the use of pine beams. This species is a popular and widely utilised material in Poland, for example, in the fabrication of components for roof trusses. The dimensions of the beams were 80 × 80 mm in cross-section and 1600 mm in length. These dimensions were determined by the characteristics of the test site (maximum length of the beam), economic considerations, and the standard [12] (height of the beam proportional to its length) used.

Material tests were carried out to classify the wood into the appropriate standard class. The EN 408 [12] standard was used to determine the mechanical properties of the wood. These parameters were determined on 5 samples. They are further described in the paper. The flexural strength of the wood fm,k was equal to 56.22 MPa (standard deviation 7.15 MPa). The global elastic modulus of the wood Em,g was equal to 9.50 GPa (standard deviation 0.82 GPa). The volumetric density of the wood determined before the reinforcement application was 538.5 kg/m^3^. This permits the wood to be categorised within Class C20, as defined by the European Standard EN 338 [13]. The low bending stiffness value should be highlighted, as it is a pivotal factor in determining the suitability of the component in question. The low stiffness and high deflection of the elements may be a contributing factor in the reinforcement of timber structures. The flexural strength is several times higher than the value specified for the given class.

Prior to the commencement of the tests, the beams were stored for a period of several years in locations that provided protection from the effects of the weather. The mean moisture content of the wood, as determined following the completion of the experimental trials (on the day the elements were bent), was found to be 9.63%. The standard deviation of the moisture content was found to be 0.16%. The measurements were obtained using a Tanel WRD-11 electrofusion moisture meter [14].

#### 2.1.2. Carbon-Fibre-Reinforced Polymer Sheet

The reinforcement of the structure was achieved through the utilisation of S&P C-Sheet 640 [15] unidirectionally reinforced carbon fibre sheets, which possess an ultra-high modulus of elasticity. The sheets were produced by S&P Reinforcement Polska, a company based in Malbork, Poland. The fundamental physical and mechanical characteristics of the reinforcement are outlined in Table 1. It is notable that the modulus of elasticity of the composite is of a value that is many times higher than that of wood. The modulus quotient is approximately 67. This parameter indicates the high potential of the material to enhance the stiffness of the wood–FRP system. One disadvantage is the low value of its elongation at failure. The relatively thin reinforcement (0.189 mm) permits the selection of an appropriate number of layers, thereby optimising the composite’s resistance to wear and tear.

**Figure 1:**
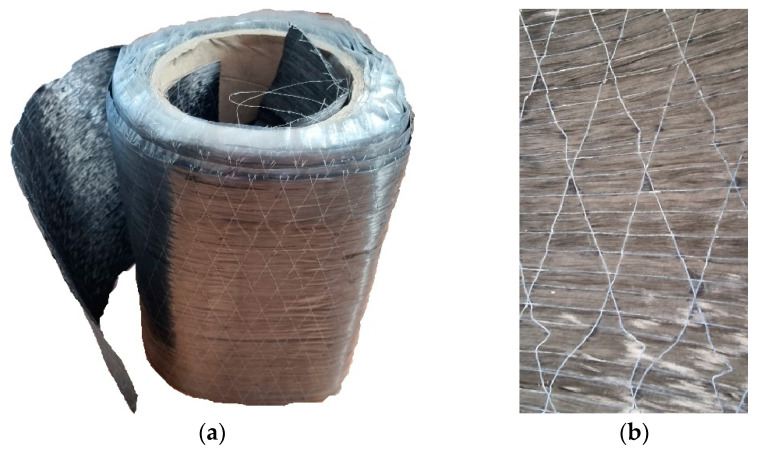
Composite reinforcement: (**a**) CFRP roll; (**b**) closer view of carbon sheet.

The sheets were delivered in a roll measuring 30 cm in width. The width of the applied sheet was equal to the width of the beam: 8 cm. The total length of the reinforcement was 140 cm. This value is equivalent to the length of the unsupported portion of the beam. The composite material was cut to size with scissors. Figure 1 illustrates a view of the sheet and a close-up of the fibres. The external surface of the sheet is covered with a protective layer of foil. The foil was removed directly before the application of adhesive. The load-bearing fibres are arranged in the main direction of the fabric, while the stabilising fibres are positioned in the transverse direction, forming a diagonal pattern. The thickness of the reinforcement was found to be 0.189 mm and 0.378 mm for one and two layers of reinforcement, respectively.

#### 2.1.3. Adhesive

A hand lay-up process was implement for reinforcement application. The sheets were adhered using a two-component epoxy-resin-based adhesive, designated as S&P Resin 55 HP [16]. The adhesive was prepared by mixing it for approximately five minutes using a slow-speed mixer until a uniform consistency was achieved. The adhesive mixture was applied to both the sheet and the wooden surface. Prior to the application of the adhesive, the wood surface was sanded with a belt sander (using 120 grit sandpaper) and cleaned. The adhesive was distributed with the aid of a rubber spatula. An excess of adhesive was applied to the upper surface of the sheet. The quantity of adhesive utilised was approximately 1 kg per square metre of sheet. The fundamental characteristics of the adhesive are presented in Table 2.

### 2.2. Methods

This research was conducted at the Laboratory of Strength of Materials at Kielce University of Technology. The objective of this research was to ascertain the feasibility of enhancing the stiffness of beams through the utilisation of a material exhibiting markedly superior characteristics. In order to achieve this objective, an investigation was conducted into the bending behaviour of unreinforced and reinforced beams. The bending conditions were implemented in accordance with the specifications set forth in PN-EN 408 [12].

**Figure 2:**
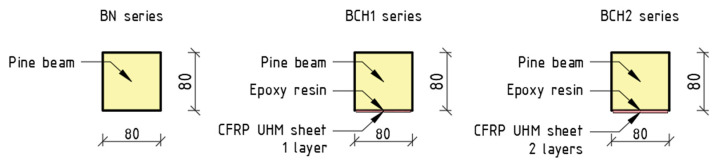
Strengthening configurations.

The bending test series, as illustrated in Figure 2, was composed of the following: BN—reference beams; BCH1—beams reinforced with a single layer of CFRP UHM sheet glued to the underside; and BCH2—beams reinforced with two layers of CFRP UHM sheet bonded one over the other to the underside. Each series (BN, BCH1, and BCH2) was composed of 5 beams. A total of 15 beams were tested (5 unreinforced and 10 reinforced). The degree of cross-sectional reinforcement was found to be 0.24 and 0.48% for the reinforced series BCH1 and BCH2, respectively. The reinforcement was confined exclusively to the tension zone.

**Figure 3:**
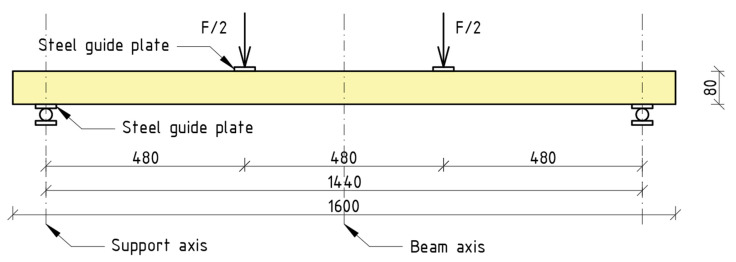
Schematic representation of test setup.

A schematic representation of the test apparatus is provided in Figure 3. The characteristic dimensions were derived through the application of a multiple of the cross-sectional height. The overall length of the span was 144 cm. The distance between the actuators was 48 cm. The load applied to the beam was distributed in a symmetrical manner.

**Figure 4:**
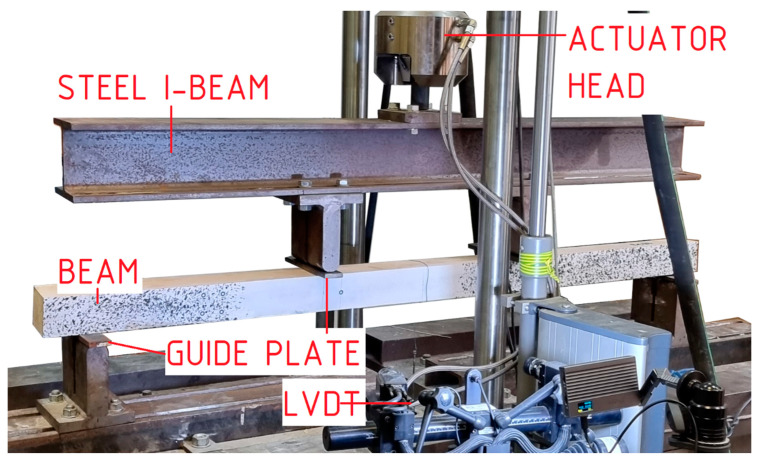
View of the test setup.

The view of the research station is shown in Figure 4. The bending tests were conducted on an MTS-322 testing machine (Shenzhen, China), which has an actuator capacity of 100 kN. The force generated by the actuator was divided into its constituent components by means of a steel crosshead. The load on the beams was regulated by establishing a constant displacement value for the actuator of the testing apparatus: 7 mm/min. The displacement speed was selected in accordance with the recommendations set forth in EN 408 [12], with the objective of ensuring that the failure of the unreinforced beams would occur within the recommended time frame. Steel guide plates, with a thickness of 1 cm and a width of 4 cm, were positioned on the supports and at the point of application of the concentrated force.

During the course of the test, the following data were recorded: the force and displacement of the actuator, the deflection of the beam at the centre of the span using a linear displacement sensor, and the duration of the test. Furthermore, lateral surface deformations were quantified utilising an ARAMIS-type optical system.

## 3. Results

The results of the analyses are presented in terms of load-carrying capacity, stiffness, bending ductility, and failure mode. Furthermore, the potential of employing numerical modelling to anticipate bending stiffness values was investigated.

### 3.1. Load-Bearing Capacity

**Figure 5:**
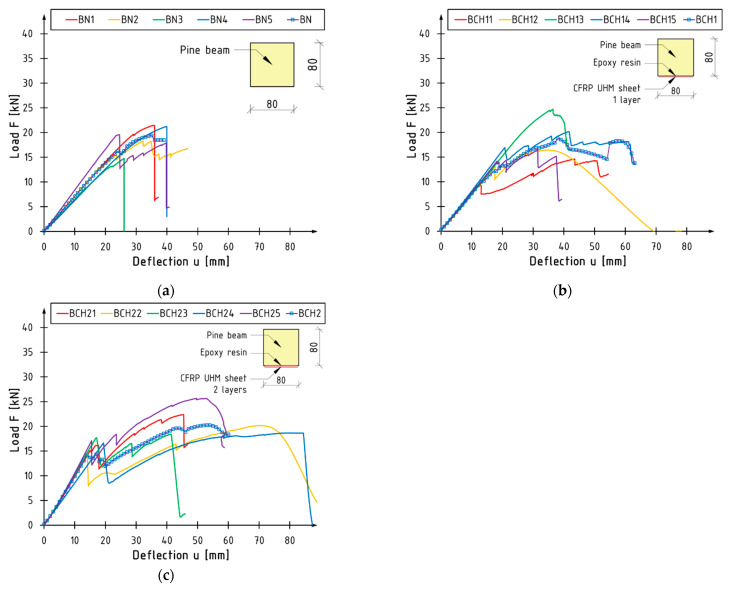
Load versus deflection curves for series (**a**) BN; (**b**) BCH1; (**c**) BCH2.

Figure 5 shows graphs of the relationship between load F and deflection u at the centre of the beam span for all the elements tested. The graphs are presented in a manner that allows for the identification of individual test series. The images illustrate the mean curve for each series (labelled BN, BCH1, and BCH2) and a cross-sectional view of the tested series. The mean value is indicated by lines with symbols (blue squares). Its course is the result of all the elements that have been tested for a given series, and it can vary significantly from one instance to the next. The occurrence of jumps is contingent upon the failure of specific elements.

In the case of unreinforced beams, the behaviour in question may be considered to be almost linear from the point of inception to the point of failure. In the case of BN1 and BN4 beams, a non-linear phase was observed in the final phase of the test: plasticisation in the compression zone occurred. The maximum deflection is typically less than that observed in reinforced beams. The point of failure is typically associated with a precipitous decline in load values resulting from the fracturing of timber.

The failure of reinforced beams occurs in two distinct stages. The data demonstrate a series of reductions in load, which are followed by a period of strengthening and an increase in load. The initial reduction in force is attributable to the rupture of the composite mat. This indicates that, in each of the analysed cases, the bending strength (maximum load value) is the responsibility of the unreinforced section, with the sheet only engaging in the initial phase of the beams. The moment of sheet rupture can be regarded as the theoretical elastic limit. This phenomenon is most clearly observed in the case of the BCH2 series. The steeper slope of the curve, indicative of increased stiffness, occurs prior to the failure of the composite. Following the drop, the slope is analogous to that of an unreinforced beam. Following the fracture of the composite reinforcement, the plastic phase of the element becomes evident. Furthermore, it can be observed that the composite reinforcement results in a reduction in work variation during the initial bending phase of the beams. The initial slope of the curves is nearly identical. The composite ruptured, resulting in localised damage to the wood.

The results of the aforementioned relationships are presented in tabular form below (Table 3, Table 4 and Table 5). They contain information on the maximum force F_max_ and the corresponding deflection value U_Fmax_. Furthermore, the principal type of failure form is indicated in accordance with the following designations: T—fracture of wood in tensile zone and RC—rupture of composite sheet. The arrow serves to indicate the sequence of the failure form.

It is important to note that there is a considerable degree of scatter in the deflection values for BCH2: a standard deviation of 16.10 mm. In contrast, the reinforcement does not significantly impact the spread of results for the other parameters. The standard deviation values are found to be comparable between the various batches.

**Figure 6:**
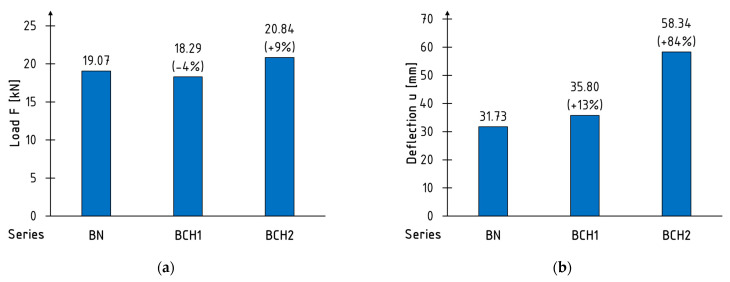
Mean values of (**a**) maximum load; (**b**) deflection at maximum load.

The mean values of the maximum force and the corresponding deflection are illustrated in Figure 6. The reinforcement did not result in a discernible impact on the augmentation of flexural load capacity. The mean value of the maximum failure force is comparable across all series. A modest increase was observed for the BCH2 series, amounting to 9%. Nevertheless, the beams of the BCH1 series exhibited a 4% decline in comparison to the BN series. This behaviour is attributable to the low elongation value at the point of composite failure. The composite failed to reach its strength before the object in question was damaged. It can be concluded that there are no notable discrepancies in the value of this parameter between the series. The load-bearing capacity of the section is that of the unreinforced wood, which is to be expected given the nature of the material. Nevertheless, it is evident that the BCH2 series beams exhibit a considerable increase in deflection when compared to unreinforced beams. This phenomenon is associated with the enhanced ductility of the beams following composite failure: the plasticisation of the section subsequent to sheet rupture. Despite the sheet rupture, it remained rigidly attached to the timber. This may indicate that, to a certain degree, the reinforcement had a restrictive impact on the tensile stresses in the shear zone.

The behaviour of the reinforced beams was anomalous, which can be attributed to the intrinsic characteristics of the reinforcing material. It is anticipated that the reinforcement of timber beams with sheets will result in an increase in load-bearing capacity by a factor of several to tens of percent, as evidenced by studies [1,4,17,18]. The same effect is observed for both small beams and full-sized elements. Also, it depends on the length of reinforcement [19].

Conversely, the effect of composite reinforcement failure is markedly distinct. It is typical for the destruction of the reinforcement to be accompanied by considerable damage to the timber and a reduction in the loading force [2,3]. Here, an additional increase in the loading force was achieved. This indicates that the load capacity remains unaffected by the addition of the composite material.

### 3.2. Bending Stiffness

**Figure 7:**
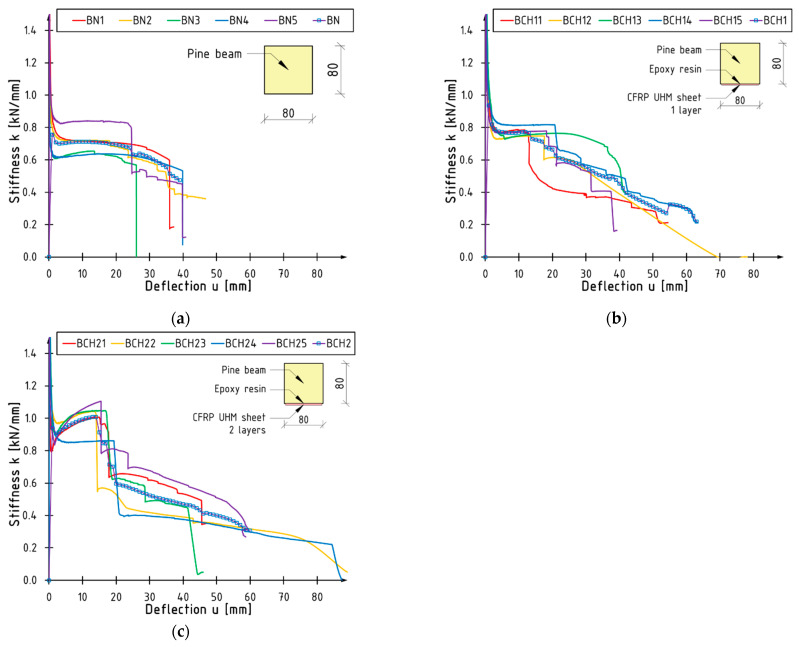
Stiffness versus deflection curves for series (**a**) BN; (**b**) BCH1; (**c**) BCH2.

The graphs illustrating the alterations in stiffness during bending are presented in Figure 7. The progressive alterations are evident. The initial, uppermost staircase is indicative of the scenario wherein the undamaged timber–CFRP UHM cross-section exhibits complete cooperation. The conclusion of the process is marked by the onset of timber cracking in the case of unreinforced beams or sheet rupture in the case of reinforced beams. Subsequent decreases in stiffness values are attributable to the cracking or plasticisation of the timber. Changes in curvature can be used for the prediction of failure, as stated in [9].

It is noteworthy that the graphs of beams reinforced with two layers of CFRP UHM mat (BCH2 series) exhibit an intriguing phenomenon. Specifically, an initial reduction in stiffness is followed by a gradual increase until the composite reaches its rupture point. This phenomenon has been observed in tests involving loads of approximately 2 to 20 kN. It seems reasonable to posit that this phenomenon is related to the composite turning on at successive sections along the length of the beam. This behaviour is anomalous and has not been previously documented in the author’s own research.

**Figure 8:**
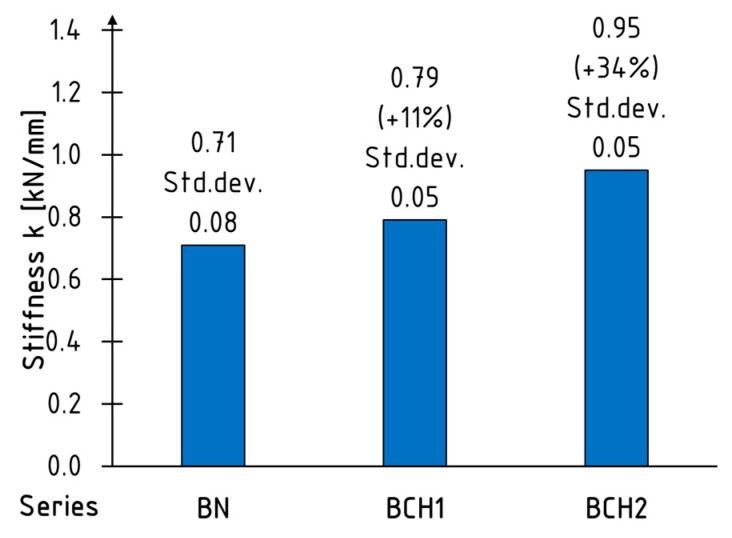
Mean values of bending stiffness coefficient.

In order to facilitate a comparison of the stiffness of the individual components, k coefficient was estimated in accordance with the formula set forth by [20]:

k = F/u,(1)

where F is the load value (in kilonewtons) and u is the deflection at the midspan (in millimetres). Given the considerable variability in stiffness during bending, the value was determined for a load range of 0.1 to 0.4 of the maximum force in order to account for this variability. The mean values for each series, along with the standard deviations, are presented in Figure 8. The increase in stiffness is 11 and 34%, respectively, for one and two layers of sheet reinforcement. It can be seen, therefore, that the relationship is not linear.

It is evident that the gains achieved are superior to those that would be attained by the use of glass, aramid, and carbon-fibre-reinforced sheets of comparable thickness [20]. For example, Basterra et al. [11] obtained an average increase in stiffness using glass sheets as a reinforcement equal to 12.1% and 14.7% for 1.07% (one layer) and 1.6% (two layer) reinforcement ratios, respectively. Given the minimal thickness of the material, it is possible to enhance this effect by incorporating additional layers of reinforcement. The positive effect of increasing the degree of cross-sectional reinforcement on the stiffness gain is demonstrated in reference [10].

### 3.3. Bending Ductility

**Figure 9:**
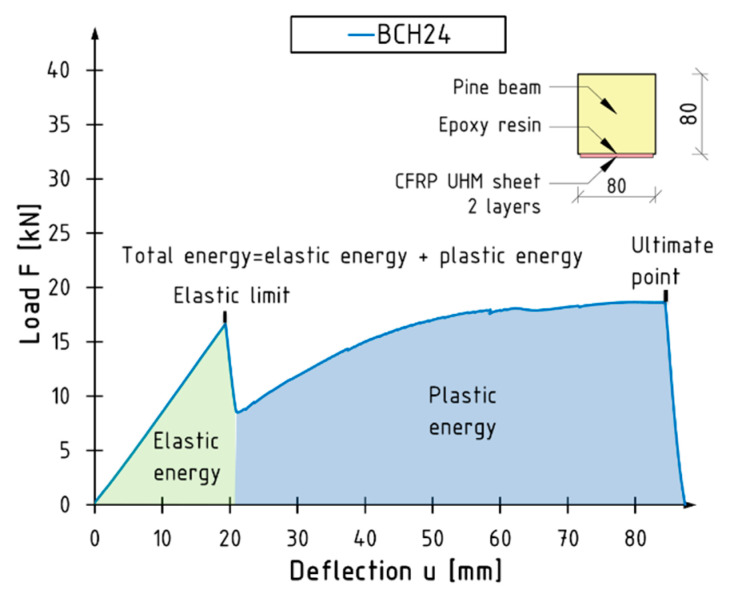
Representation of elastic, plastic, and total energies.

An index based on energy absorption µ_E_ was used to determine the ductility of the flexural beams. This index was determined through the following formula [20]:

(2) $$\mu_{E}=\frac{1}{2}\cdot \left(\frac{W_{\mathrm{tot}}}{W_{el}}+1\right),$$

where W_el_ is the elastic energy (fraction of total) of the area under the load–deflection curve up to elastic limit and W_tot_ is the total energy of the area under the load–deflection curve up to failure. The elastic and total energies were estimated by integrating polynomials that describe the load–deflection relationship. The boundaries of the integration were defined by the characteristic points on the diagrams, including the starting point, the elastic limit, and the moment of failure of the beam. The following assumptions were made in order to facilitate the calculations: (1) the linear–elastic range occurred from the start of the test until the composite reinforcement broke (elastic limit) or a load equal to 0.7 F_max_ was reached for unreinforced beams and (2) the plastic range was followed. In instances where substantial alterations were made to the course, polynomial gluing was employed in the relevant ranges. The calculations were performed using the software programmes MathCad 15 and Excel 2016. Figure 9 illustrates a section of a reinforced beam curve from a two-dimensional perspective.

Table 6 presents the mean values of elastic energy, total energy, and ductility index. The elastic working range for the reinforced beams was found to be significantly shorter. The mean elastic energy of the BCH1 and BCH2 series was found to be in excess of 30% lower than that of the BN series beams. This is connected to the alteration in the slope factor of the curves within the elastic range. Nevertheless, as the degree of cross-sectional reinforcement augmented, the plastic working range and, thus, the ductility index augmented in parallel. The maximum relative increase observed for the BCH2 series beams was 248% in comparison to unreinforced beams.

Similar values of ductility (based on deformation analysis), equal to 240.7% when compared to unreinforced beams, were obtained by Mansour et al. [21] in numerical studies. However, that value corresponded to a scenario when a CFRP sheet was applied in tensile and compressive zones at the same time.

### 3.4. Failure Modes

**Figure 10:**
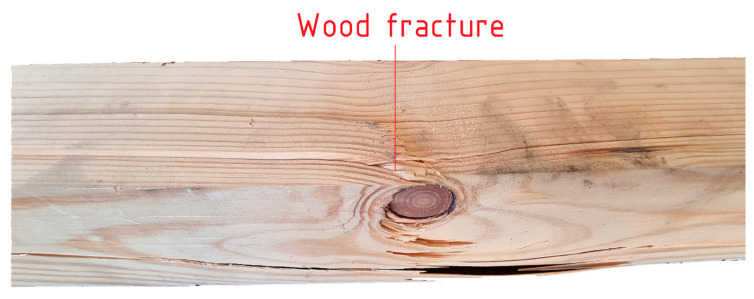
Typical failure mode of unreinforced beam (T).

The failure of the non-strengthened beams was caused by the fracture of the timber in the tensile zone (failure mode marked earlier in the paper as T). The fracture was unanticipated and abrupt. The onset of failure occurred in the zone of maximum bending moment: tensile strength parallel to the grain was exceeded. As illustrated in Figure 10, the fracture originated within the knot. The cracking was accompanied by a loud sound.

**Figure 11:**
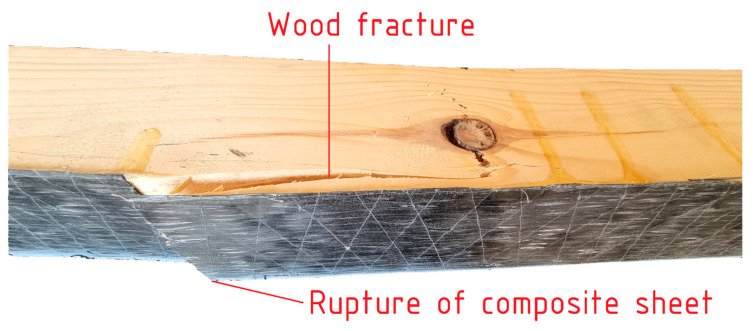
Typical failure mode of reinforced beam (RC → T).

Reinforced beams were destroyed in two phases: (1) rupture of the composite sheet resulting from reaching the elongation limit at composite rupture (failure mode marked earlier in the paper as RC) (2) followed by wood cracking in the tension zone analogous to unreinforced beams. Figure 11 illustrates an instance of failure in a beam reinforced with a single layer of CFRP UHM mat. The mat break is positioned at a right angle to the longitudinal axis of the beam: it is typical. The phenomenon of timber cracking is typically initiated at the point of rupture in the mat. As illustrated in the example, the crack progressed to the nearest timber defect. The ripping of the sheet was accompanied by a single loud sound, which was followed by multiple quieter sounds corresponding to the crushing and cracking of the wood.

The failure forms reported in the literature are typical of both unreinforced beams and beams reinforced with composite sheets with minimal cross-sectional reinforcement [1,2,3,4]. It needs to be pointed out that the amount of reinforcement can significantly influence the failure modes [22]. Nevertheless, the application of CFRP as a reinforcement increases the maximum values of deformations and prevent the development of cracks [23].

### 3.5. Numerical Analysis

**Figure 12:**
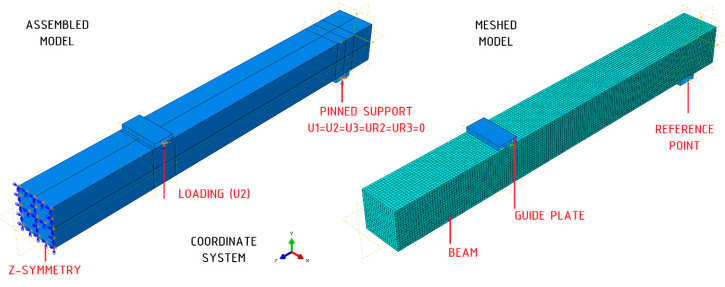
View of numerical model.

The standard module of the Abaqus 2017 software was employed for the purpose of carrying out numerical simulations [24]. In consequence of the symmetry of the system, only half of the element was constructed. The beams were modelled as three-dimensional deformable bodies. The cross-section was extruded to a length of 800 mm. The composite was taken into consideration as a sheet within three-dimensional space. In contrast, the steel guide plates were represented as non-deformable bodies, characterised as non-bendable (analytical rigid bodies). In order to analyse the behaviour of the wood, a linear perfectly plastic material model was adopted. The CFRP sheet was modelled as linear elastic. The material properties are discussed in detail in Section 2.1.1 and Section 2.1.2. The process of meshing was conducted on the aforementioned components. In the case of the beams, an element of the C3D8R (8-node brick element) type was assumed. In the case of the composite, an element type S4R (4-node shell element) was posited as the most probable hypothesis. In this study, the mesh size of the finite element mesh was assumed to be 5 mm. The bonding between the composite and the timber was modelled as an ideal bond, utilising a “tie” type of connection. At the same time, the impact of the adhesive was omitted; no instances of detachment failure were recorded in the experimental studies. In order to account for the symmetry of the system, a Z-SYMM bond was introduced on the face plane. Contact was considered through the use of a surface-to-surface contact option. In consideration of the inability of the body to penetrate in the normal direction and a friction coefficient of 0.3, the contact properties were evaluated. The analyses were conducted within the static range. The loading was achieved by displacing a steel guide plate on the upper surface. A representation of the numerical model is presented in Figure 12.

**Figure 13:**
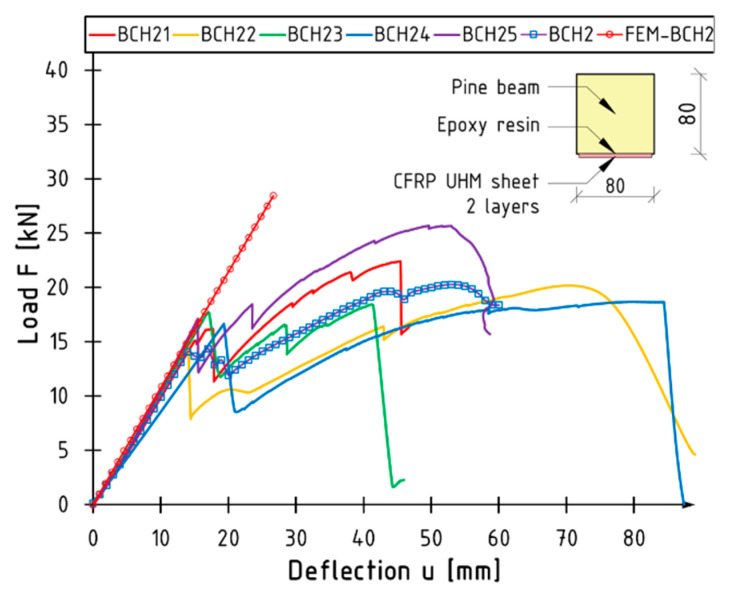
Numerical versus experimental curves.

The outcome of the numerical tests is illustrated with the aid of the BCH2 series beams as a case in point. Figure 13 illustrates the curve derived from the numerical model (FEM-BCH2) in comparison to the other results. A highly satisfactory agreement was obtained within the linear–elastic range. Nevertheless, the model does not incorporate the fracture of the composite material or the alteration in the gradient of the curve. In the other series, the convergence of the results was comparable. The discrepancy between the experimental and numerical bending stiffness values was within a few percent.

### 3.6. Theoretical Analysis

The equivalent cross-section method was also employed for the estimation of theoretical bending stiffness values. The methodology is illustrated through its application to the prediction of the behaviour of reinforced timber beams, as detailed in reference [25]. Furthermore, the methodology was successfully employed to estimate the mechanical properties of glued laminated timber beams with varying parameters of the individual layers [26]. The assumptions of the method are described in detail in [27,28]. Here, only the main assumptions were provided.

The position of the neutral axis (zc) was evaluated according to Formula (3):

(3) $$zc=\frac{Ai\times zi}{\sum_{i=1}^{n}Ai},$$

where Ai is the cross-section area of field; zi is the distance from the centroid of field to horizontal axis; and i is the number of field. The position of the horizontal axis (datum) was assumed to be aligned to the bottom part of the section.

The moment of inertia of the transformed cross-section (Itransformed) was evaluated according to Formula (4):

(4) $$Itransformed=\sum_{i}^{n}\left(Iyi+Ai\times {\left(zc-zi\right)}^{2}\right),$$

where Iyi is the second moment of inertia of the field.

Bending stiffness was evaluated for both unreinforced and reinforced sections as a product of the modulus of elasticity of wood and the moment of inertia of the section. In this case, the bending stiffness of the beam can be estimated using the following Formula (5):

(5) $$Bending\;stifness=\left\{\begin{array}{cc} {EI}_{Transformed}, & \varepsilon <\varepsilon f\\ EI, & \varepsilon \geq \varepsilon f \end{array}\right.,$$

where E is the modulus of elasticity of the wood (from point 2.1.1), I is the moment of inertia of the unreinforced beam, Itransformed is the moment of inertia of the transformed section (Equation (4)), ε is the strain of the composite; and εf is the limiting strain in the composite (from Table 1). In accordance with the aforementioned considerations, a bilinear graph of the load–deflection relationship will be obtained.

The theoretical value of midspan deflection can be accurately evaluated from Formula (6):

(6) $$u=\frac{F\times a}{24\times \left\{\begin{array}{c} EI\;\;\;for\;unreinforced\;section\\ EItransformed\;for\;reinforced\;section \end{array}\right.}\times \left({3\times L}^{2}-{4\times a}^{2}\right),$$

where F is half of the loading force; a is the distance between the support and the nearest loading axes; and L is the span length. Equation (6) is suitable for the linear relationship between force and deflection. Therefore, it is not able to predict post-elastic deformations after the rupture of the composite sheet.

**Figure 14:**
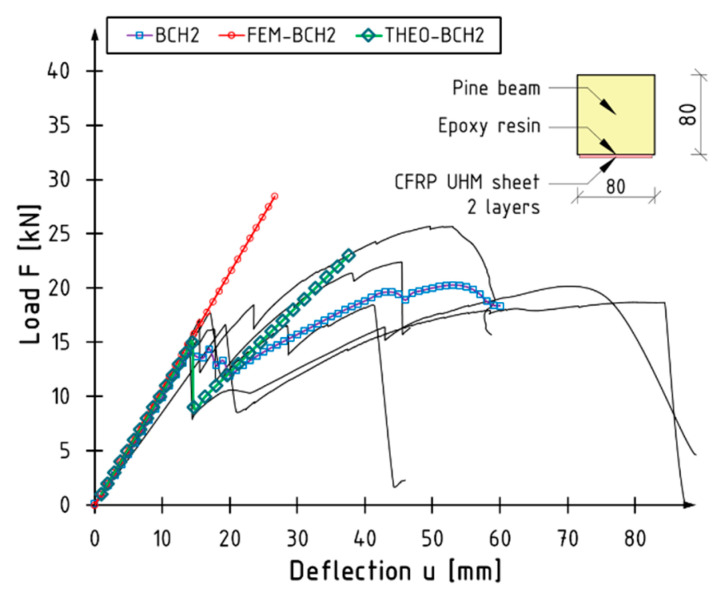
View of modified theoretical curve (THEO-BCH2).

The authors propose that the curvature of the beam, which will be used to estimate the deformation of the external surfaces, should be employed to make a preliminary determination of the suitability of the material for reinforcement. It can be assumed that the deformation of the composite will be equal to that of the wood surface. The point at which the slope undergoes a change is when the limit strain in the composite is reached. The gradients of the individual curves are derived from the characteristics of the transformed or wood cross-section. It is evident that such a continuation will not align with the actual work. Through the implementation of suitable alterations, it is feasible to procure a more precise iteration of the graph in the subsequent phase. As a consequence of the aforementioned modifications, the second curve is shifted down to a point that corresponds to the theoretical position of the load–deflection relationship for an unreinforced beam, without the consideration of reinforcement. The aforementioned simplifications should facilitate an initial estimation of the suitability of the material type. Figure 14 illustrates the outcomes of this methodology for BCH2 series beams. The green colour with markers indicates the modified bilinear curve (THEO-BCH2). The model accurately predicts the failure moment of the composite and the load force fault. One limitation of this approach is that it does not consider the potential plasticisation of the wood and the associated risk of failure. Nevertheless, the predicted initial beam stiffness and the point of rupture of the composite corresponds well with the experiment.

The results of the theoretical stiffness analysis (stiffness coefficient k evaluated according to Equation (1)) compared with experimental and numerical ones are presented in Table 7. In the case of unreinforced beams, the theoretical stiffness values were found to be slightly lower. In the case of reinforced elements, the proportion was found to be reversed. The highest difference was equal to 13%. Those differences could be associated with the variability of the mechanical properties of wood.

As with the numerical analysis, a high degree of convergence was observed between the theoretical and experimental stiffness values. Nevertheless, the estimated value for the BCH1 series was somewhat in excess of the actual figure. The observed increases are characterised by a linear variation.

Using Equations (3)–(6), theoretical analysis was performed to predict the bending stiffness of beams reinforced with different composite materials. The glass-fibre-reinforced polymer (GFRP), aramid-fibre-reinforced polymer (AFRP), and high-strength-carbon-fibre-reinforced polymer (CFRP) sheets were considered as possible reinforcement. The material properties of each sheet type are provided in Table 8.

The reinforcement configuration was the same as described earlier in the paper: the composite sheet bonded to the bottom surface. Different numbers of reinforcement layers were investigated (up to 3). The results of the analysis are presented in Table 9. The obtained increases were linear for every material used. Similarly, the lowest reinforcement effectiveness was achieved for the AFRP and GFRP sheet. The highest bending stiffness EI values were obtained for the beam reinforced with CFRP UHM, which showed percentage increases approximately four times higher than those achieved with AFRP and GFRP. The application of high-strength CFRP brought intermediate results.

## 4. Conclusions

This paper presents an analysis of the static working behaviour of unreinforced and sheet-reinforced CFRP UHM timber beams. The results demonstrated that the method was effective in improving both the stiffness and the ductility of the beams. An 11% and 34% increase in stiffness coefficient was achieved for beams reinforced with one and two layers of composite, respectively. The maximum increase in ductility was 248% for BCH2 series when compared to unreinforced beams. Furthermore, the reinforcement resulted in a reduction in the scatter of bending stiffness values. Nevertheless, the reinforcement did not result in a significant increase in the load-carrying capacity. For example, the BCH1 series average value of the maximum loading force was 4% lower than for the BN series. Only a 9% increase in the average value of loading force was obtained for the BCH2 series. The load-bearing capacity of the beams was contingent upon the strength of the timber employed.

Both the numerical and mathematical model were found to be useful in predicting the bending behaviour of unreinforced and reinforced beams within the elastic range. The maximum relative error was equal to 13%. However, due to their simplicity, they cannot be used to describe post-elastic work.

It was demonstrated that one of the constraints on the utilisation of composites is the value of elongation at failure. In the case of timber beams, the curvature of the beams and the corresponding strain value of the composite can be employed as a control parameter. Using them, the moment of failure of the composite can be predicted.

This work is constrained by a number of limitations. These apply to both the dimensions that were tested and the materials that were used. Furthermore, the test was conducted under static loading conditions within a predefined static scheme. The parameters that have been identified are ad hoc and not subject to external influences. It is possible that the aforementioned conditions may differ significantly from the actual operating conditions of the objects that were constructed within the facility.

## Figures and Tables

**Table 1:** Selected physical and mechanical parameters of composite reinforcement (based on [15]).

Parameter	Value
Modulus of elasticity Ef [GPa]	640
Tensile strength ft,f [MPa]	2600
Density ρf [kg/m^3^]	2120
Elongation at rupture εf [%]	0.4

**Table 2:** Selected physical and mechanical parameters of epoxy resin (based on [16]).

Parameter	Value
Bending strength fm [MPa]	85.3
Modulus of elasticity Ek [MPa]	3200
Density ρk [kg/m^3^]	1200–1300
Compressive strength fc,k [MPa]	100

**Table 3:** Results for BN series.

Parameter	Beam					Mean Value (Std. Dev.)
	BN1	BN2	BN3	BN4	BN5	
*F_max_* [kN]	21.471	18.33	14.80	21.23	19.50	19.07 (2.42)
*U_Fmax_* [mm]	35.93	32.24	26.04	39.88	24.56	31.73 (5.80)
Failure mode	T	T	T	T	T	-

**Table 4:** Results for BCH1 series.

Parameter	Beam					Mean Value (Std. Dev.)
	BCH11	BCH12	BCH13	BCH14	BCH15	
*F_max_* [kN]	14.62	15.43	24.71	20.18	16.51	18.29 (3.73)
*U_Fmax_* [mm]	43.51	25.90	36.39	41.69	31.49	35.80 (6.50)
Failure mode	RC → T	RC → T	RC → T	RC → T	RC → T	-

**Table 5:** Results for BCH2 series.

Parameter	Beam					Mean Value (Std. Dev.)
	BCH21	BCH22	BCH23	BCH24	BCH25	
*F_max_* [kN]	22.38	19.12	18.32	18.70	25.70	20.84 (2.82)
*U_Fmax_* [mm]	45.18	75.77	41.42	79.71	49.64	58.34 (16.10)
Failure mode	RC → T	RC → T	RC → T	RC → T	RC → T	-

**Table 6:** Mean values of ductility parameters.

Series	W_el_ [J]	W_tot_ [J]	µ_E_ [-]
BN	209.42	390.65	1.46
BCH1	132.14 (−37%)	525.95	2.72 (+186%)
BCH2	138.55 (−34%)	842.68	3.62 (+248%)

**Table 7:** Comparison of different values of stiffness coefficient k.

Series	Stiffness Coefficient k [kN/mm]		
	Experimental	Numerical	Theoretical
BN	0.71	0.65 (−8%)	0.62 (−13%)
BCH1	0.79	0.88 (+11%)	0.85 (+7%)
BCH2	0.95	1.07 (+13%)	1.04 (+9%)

**Table 8:** Material properties of composite sheets (based on [29]).

Parameter	AFRP Sheet	GFRP Sheet	CFRP Sheet
Modulus of elasticity Ef [GPa]	120	65	265
Tensile strength ft,f [MPa]	2900	2850	5100
Thickness tf [mm]	0.200	0.299	0.222

**Table 9:** Predicted values of bending stiffness.

No. of Layers	Bending Stiffness EI [kNm^2^]				
	Unreinforced Beam	Beam Reinforced with			
		AFRP Sheet	GFRP Sheet	UHM CFRP	CFRP Sheet
1	32.427	35.238 (+8%)	35.072 (+8%)	45.117 (+39%)	39.045 (+20%)
2		37.919 (+17%)	37.618 (+16%)	54.997 (+70%)	44.894 (+38%)
3		40.482 (+25%)	40.073 (+24%)	62.936 (+94%)	50.110 (+55%)

## Data Availability

The original contributions presented in this study are included in the article. Further inquiries can be directed to the corresponding author.
